# Association between dietary consumption of multiple vitamins and age-related macular degeneration: a cross-sectional observational study in the National Health and Nutrition Examination Survey 2005–2008

**DOI:** 10.3389/fnut.2024.1504081

**Published:** 2024-11-11

**Authors:** Zhao Liu, Qiuyuan Wang, Lu Li, ShanJun Cai

**Affiliations:** ^1^Aier Eye Hospital of Guizhou Province, Guiyang, China; ^2^Zunyi Medical University, Zunyi, China; ^3^Guizhou Branch of the Affiliated Hospital of Zunyi Medical University, National Clinical Research Center of the Eye Hospital of Guizhou Province, Key Laboratory of Eye Disease Characteristics of Guizhou Province, Zunyi, China

**Keywords:** vitamin, macular degeneration, NHANES, causality, epidemiology, dietary intake

## Abstract

**Purpose:**

Age-related macular degeneration (AMD) is one of the common causes of blindness in the elderly worldwide. Its prevention and monitoring indicators remain a key area of research. This study aims to examine the association between vitamin intake and AMD prevalence.

**Methods:**

Data from the National Health and Nutrition Examination Survey (NHANES) from 2005 to 2008 were used for cross-sectional analysis. Logistic regression models, subgroup analyses and multicollinearity regression were employed to assess the association between vitamin intake and AMD.

**Results:**

A total of 1,627 participants were included, with 54.5% (weighted) males and 45.5% (weighted) females. Significant differences were observed in the intake of vitamins B (B1, B2, B6, and B12), E, and folic acid between the AMD and Non-AMD groups. The Non-AMD group had higher average intakes (weighted) of vitamin B1 (1.71 ± 1.10 vs. 1.37 ± 0.64), B2 (2.42 ± 1.22 vs. 1.86 ± 0.70), B6 (2.05 ± 1.25 vs. 1.71 ± 0.85), B12 (5.73 ± 6.18 vs. 4.54 ± 3.27), E (7.93 ± 5.47 vs. 6.39 ± 2.86), and folic acid (181.87 ± 178.04 vs. 140.72 ± 124.60). Logistic regression and subgroup analyses further supported these findings.

**Conclusion:**

This study found that higher vitamin intakes B and E were associated with a lower prevalence of AMD in the U.S. population. Eating a healthy diet rich in vitamins B and E, particularly B2 (eggs, green vegetables, meat, mushrooms, and almonds) may help to reduce vision loss due to AMD. However, since this is a cross-sectional study, causal associations between vitamin intake and AMD cannot be established. Further randomized clinical trials are needed to confirm these findings.

## 1 Introduction

Age-related macular degeneration (AMD) is the common causes of low vision and blindness among the elderly (1). Its current global prevalence is approximately 8.69% among individuals aged 45–85 years, and the number of affected individuals is expected to rise to 288 million by 2040 (2). Consequently, the study and prevention of AMD risk factors have become a significant focus in clinical research. This disease arises from a complex interplay of aging, genetic predispositions, and environmental influences, including dietary habits (3–5). Late AMD presents in two forms: geographic atrophy (GA) and neovascular AMD. Neovascular AMD necessitates repeated intravitreal injections of anti-vascular endothelial growth factor (anti-VEGF) drugs, whereas there are currently no clinical treatments available that can either slow progression or restore vision in cases of GA (2). Therefore, effective prevention and detection methods are crucial for mitigating AMD-induced blindness in the elderly population. Oral administration of a specific combination of antioxidants and mineral supplements has been shown to slow the progression from intermediate to late AMD, particularly in cases of neovascular AMD (6, 7). However, some observational studies have indicated that high intakes of specific dietary nutrients are associated with either a reduced or increased risk of early or late AMD (8, 9), although the findings remain inconsistent. Over the past few decades, research has predominantly centered on common factors influencing the development of AMD, such as age, gender, smoking, alcohol consumption, hypertension, diabetes, genetic factors, and environmental influences (10, 11). Recently, dietary factors have attracted increasing attention. Some studies have suggested that oral supplements containing certain nutrients can prevent the onset of AMD or delay its progression to intermediate or late stages, particularly in neovascular cases (7, 9). A growing body of observational studies has reported that higher intake of certain dietary components (such as vitamins A, B6, B12, C, and E) correlates with decreased or increased risks of early or late AMD (12–14). In addition, some studies have found that eating a healthy diet rich in folic acid (leafy vegetables, fruits, whole grains) may help to reduce vision loss due to advanced forms of AMD (15). Although the association between vitamins and AMD has been extensively investigated, the results remain inconclusive (4, 16–20). This study used a cross-sectional design to examine the association between dietary vitamin intake and AMD. Data were drawn from the National Health and Nutrition Examination Survey (NHANES), conducted by the National Center for Health Statistics (NCHS) under the U.S. Centers for Disease Control and Prevention (CDC). The analysis focused on the association between vitamin intake and AMD in a nationally representative U.S. population sample.

## 2 Materials and methods

### 2.1 Data source

The data utilized in this study were derived from a national population study known as the NCHS, which is conducted annually by the CDC to assess the overall health of the U.S. population. The NHANES includes a series of cross-sectional interviews and examinations targeting the civilian, noninstitutionalized population of the United States. This extensive survey includes both a physical examination and a detailed interview questionnaire designed to gather information on various health issues, medications, and dietary habits. Data released from NHANES for the years 2005 to 2008 included graded fundus photographs of individuals aged 40 years and older, as well as calculated nutrient intakes based on comprehensive interviews regarding the participants’ use of dietary supplements. The large, nationally representative sample allowed researchers to investigate the association between the intake of vitamins (A, B1, B2, B6, B12, C, E, and K) and folic acid, as measured by self-reported dietary supplements, and AMD diagnosis. AMD was assessed through fundus photography. Because AMD screening and assessment were available only during the 2005–2006 and 2007–2008 periods, and because data on vitamin intakes A, B1, B2, B6, B12, C, E, K and folic acid were obtainable during these same years, this study employed the publicly available data from the 2005–2008 NHANES administration to evaluate the association between AMD and vitamin intake. Individuals aged 40 years and older represent the target demographic for AMD-related screening and, therefore, were the primary focus of this research. Ethical approval and consent were not required for this study, as it was based on publicly available data. All NHANES participants provided written informed consent to participate, and the procedures were approved by the Research Ethics Review Board of the National Center for Health Statistics. The NHANES employs a stratified multistage sampling method, necessitating a weighting mechanism to ensure the most accurate estimates of prevalence within the U.S. population. All statistical analyses conducted in this study relied on these weighted data.

### 2.2 Variables

Vitamin intakes A, B1, B2, B6, B12, C, E, and K, along with folate, were assessed through dietary interviews conducted from 2005 to 2008 and were utilized as independent variables in this study. Vitamin intake was evaluated using two 24-h dietary recalls. The initial recall interview was performed face-to-face, whereas information from the second interview was gathered via telephone, 3 to 10 days following the first assessment. Due to significant missing and unreliable data from the second follow-up, only data from the initial interview were included in the analysis. Vitamin A, K, B12, and folic acid were measured in micrograms (mcg), whereas vitamins B1, B2, B6, C, and E were measured in milligrams (mg). Dietary recall interviews were conducted by trained interviewers using an automated multipass method developed by the United States Department of Agriculture (USDA) (21). Food and beverage intake was converted to nutrient intake utilizing the USDA’s Food and Nutrient Dietary Studies Database (FNDDS), which applies data from the USDA’s National Nutrient Database for Standard Reference (NNDSR) to obtain food composition information (22). The FNDDS and NNDSR databases vary for each NHANES survey cycle. For this study, the FNDDS3 food composition database appendix for vitamins A, B1, B2, B6, B12, C, E, and K, as well as folic acid, was employed to calculate the vitamin content of foods consumed during the 2005 to 2008 cycle (23). Further information is accessible at the following URLs: https://wwwn.cdc.gov/Nchs/Nhanes/2005-2006/DR1TOT_D.htm and https://wwwn.cdc.gov/Nchs/Nhanes/2007-2008/DR1TOT_E.htm.

The diagnosis of AMD was established through fundus photography within the NHANES framework. Retinal photographs were captured for individuals aged 40 years and older from 2005 to 2008 using a digital ophthalmic imaging system (CR6-45NM; Canon USA, Inc.) and a digital camera (EOS 10D; Canon USA, Inc.). Each fundus photograph was assessed at the University of Wisconsin-Madison and graded using a classification system based on a modified version of the Wisconsin AMD Grading System. Evaluation of each photograph was performed by at least two experienced assessors, with a third senior assessor consulted in case of discrepancies.

### 2.3 Confounding factors

Potential confounding variables (covariates) were incorporated in this study to elucidate their possible effects on the association between vitamin intake and AMD. The selected covariates were based on known risk factors for AMD identified in prior research. According to the Evidence-Based Guidelines for the Diagnosis and Treatment of AMD in China (2023) and the Age-Related Eye Disease Studies 1 and 2, the following covariates are recognized risk factors for the development of AMD (2, 5): age, body mass index (BMI), hypertension, diabetes, hyperlipidemia, smoking, and alcohol consumption. Continuous variables in our analysis included age (in years) and BMI. Categorical variables included gender (male or female), race (Non-Hispanic White/Non-Hispanic Black/Mexican American/Other Hispanic/other race), education level (some college or AA [Associate of Arts] degree/high school graduate [including GED, General Educational Development], college graduate or above, 9–11th grade [which includes 12th grade without a diploma], less than 9th grade), hypertension (no/yes), diabetes (no/yes/borderline), hyperlipidemia (no/yes), smoking frequency (every day/some days/not at all), and drinking frequency (week/month/year). Detailed measurement procedures for these variables can be accessed in the public document available at https://www.cdc.gov/nchs/nhanes/. R software was utilized to address any missing covariate data.

### 2.4 Statistical analysis

All analyses employed sampling weights generated from a stratified, multistage probability sampling design in accordance with guidance provided by the NHANES. Confounding variables were selected based on findings from previous studies (2, 5). Continuous data were presented using means and standard errors of the mean (SE), whereas categorical data were described using numbers and weighted percentages. The weighted Chi-square test was applied for categorical variable comparisons between groups, and the weighted t-test was used for assessments of continuous variables. A weighted logistic regression model, taking into account various potential confounders, was utilized to investigate whether vitamin intake is independently associated with AMD. Model 1 did not include any covariates; Model 2 incorporated demographic adjustments such as age, sex, race, and education; and Model 3 enhanced Model 2 by adding adjustments for BMI, smoking status, alcohol consumption, hypertension, diabetes, and hyperlipidemia. To mitigate the mutual influence between each vitamin and the confounding factors, this study performed multicollinearity regression analysis. Data analysis was executed using R (version 4.3.2).

## 3 Results

### 3.1 Baseline analysis

**Figure 1:**
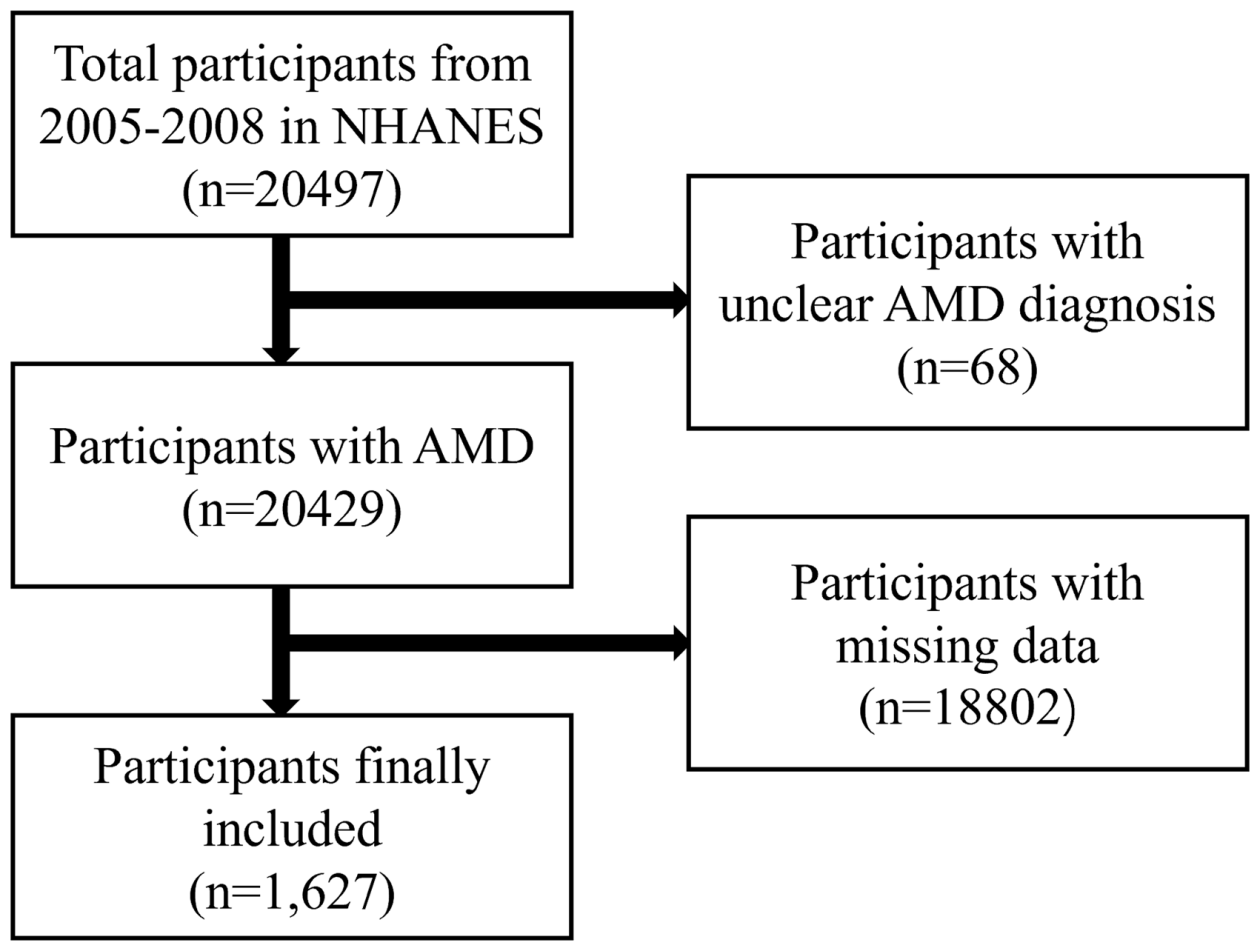
Flowchart of sample selection from NHANES 2005–2008.

Table 1 presents the demographic baseline characteristics of the study participants. A total of 20,497 individuals participated in the NHANES study from 2005 to 2008. Of these, 68 participants with unclear AMD diagnoses and 18,802 with missing data were excluded (Figure 1). Ultimately, 1,627 participants were included in this study, comprising 54.5% males and 45.5% females (weighted). The average age of the participants was 57.31 ± 11.45 (weighted) years. The participants were divided into two groups based on whether they were informed of an AMD diagnosis: the AMD group included 70 participants, whereas the Non-AMD group included 1,557 participants. The findings indicated statistically significant differences in the intake of vitamins B (B1, B2, B6, and B12), E, and folic acid between the AMD and Non-AMD groups. The average intake (weighted) for vitamin B1 was higher in the Non-AMD group compared to the AMD group (1.71 ± 1.10 vs. 1.37 ± 0.64), as well as for vitamin B2 (2.42 ± 1.22 vs. 1.86 ± 0.70), B6 (2.05 ± 1.25 vs. 1.71 ± 0.85), B12 (5.73 ± 6.18 vs. 4.54 ± 3.27), vitamin E (7.93 ± 5.47 vs. 6.39 ± 2.86), and folic acid (181.87 ± 178.04 vs. 140.72 ± 124.60). Moreover, statistically significant differences in age and smoking frequency were observed between the two groups (*p* < 0.05) (Table 1).

**Table 1:** Baseline analysis (weighted).

Variables		Overall	No	Yes	*P*
*N*		71776735.1	69346173.6	2430561.4	
Gender (%)	Female	32678120.8 (45.5)	31479286.1 (45.4)	1198834.7 (49.3)	0.54
	Male	39098614.2 (54.5)	37866887.5 (54.6)	1231726.7 (50.7)	
Age [mean (SD)]		57.31 (11.45)	56.88 (11.24)	69.46 (10.85)	<0.001
Race (%)	Mexican American	2593806.3 (3.6)	2546785.5 (3.7)	47020.8 (1.9)	0.37
	Non-Hispanic Black	5132707.3 (7.2)	4997136.3 (7.2)	135571.0 (5.6)	
	Non-Hispanic White	59736739.0 (83.2)	57550321.2 (83.0)	2186417.8 (90.0)	
	Other Hispanic	1510143.4 (2.1)	1498632.6 (2.2)	11510.8 (0.5)	
	Other race—including Multi-racial	2803339.1 (3.9)	2753298.1 (4.0)	50041.0 (2.1)	
Education (%)	9-11th grade	6486070.9 (9.0)	6229600.7 (9.0)	256470.2 (10.6)	0.95
	College graduate or above	20508353.6 (28.6)	19884829.8 (28.7)	623523.8 (25.7)	
	High school grade	18050949.5 (25.1)	17437884.7 (25.1)	613064.7 (25.2)	
	Less than 9th grade	3106780.5 (4.3)	3027555.7 (4.4)	79224.8 (3.3)	
	Some college or AA degree	23624580.6 (32.9)	22766302.7 (32.8)	858277.9 (35.3)	
Drink (%)	Month	15028290.4 (20.9)	14626932.9 (21.1)	401357.5 (16.5)	0.65
	Week	35668954.3 (49.7)	34446654.4 (49.7)	1222299.9 (50.3)	
	Year	21079490.3 (29.4)	20272586.3 (29.2)	806904.0 (33.2)	
BMI [mean (SD)]		28.76 (6.06)	28.82 (6.08)	27.12 (5.05)	0.008
Hypertension (%)	No	40268707.9 (56.1)	39212317.5 (56.5)	1056390.4 (43.5)	0.07
	Yes	31508027.1 (43.9)	30133856.1 (43.5)	1374171.0 (56.5)	
Hyperlipidemia (%)	No	35354673.8 (49.3)	34424990.1 (49.6)	929683.7 (38.2)	0.06
	Yes	36422061.3 (50.7)	34921183.5 (50.4)	1500877.7 (61.8)	
Diabetes (%)	Borderline	1498973.7 (2.1)	1432423.6 (2.1)	66550.1 (2.7)	0.74
	No	63926616.1 (89.1)	61719861.4 (89.0)	2206754.7 (90.8)	
	Yes	6351145.3 (8.8)	6193888.6 (8.9)	157256.7 (6.5)	
Vitamin_E [mean (SD)]		7.88 (5.41)	7.93 (5.47)	6.39 (2.86)	0.001
Vitamin_A [mean (SD)]		640.85 (510.64)	643.23 (514.33)	572.93 (387.48)	0.16
Vitamin_B1 [mean (SD)]		1.70 (1.09)	1.71 (1.10)	1.37 (0.64)	<0.001
Vitamin_B2 [mean (SD)]		2.40 (1.21)	2.42 (1.22)	1.86 (0.70)	<0.001
Vitamin_B6 [mean (SD)]		2.04 (1.24)	2.05 (1.25)	1.71 (0.85)	0.001
Folic_acid [mean (SD)]		180.47 (176.63)	181.87 (178.04)	140.72 (124.60)	0.04
Vitamin_B12 [mean (SD)]		5.69 (6.11)	5.73 (6.18)	4.54 (3.27)	0.007
Vitamin_C [mean (SD)]		84.27 (90.52)	83.80 (90.89)	97.54 (78.55)	0.33
Vitamin_K [mean (SD)]		104.42 (132.53)	104.11 (131.19)	113.21 (167.12)	0.65
Smoke (%)	Every day	21799007.9 (30.4)	21329402.6 (30.8)	469605.3 (19.3)	0.007
	Not at all	46894604.2 (65.3)	44933648.1 (64.8)	1960956.1 (80.7)	
	Some days	3083123.0 (4.3)	3083123.0 (4.4)	0.0 (0.0)	

AMD: Age-related Macular Degeneration, SD: Standard Deviation, CI: Confidence interval.

### 3.2 Logic analysis

To further assess the robustness of the results, a logistic regression analysis was performed after categorizing B vitamins and vitamin E into quartiles, using the Q1 group as a control. The Q4 group for vitamin B1 exhibited a negative correlation with AMD in model 1, showing a statistically significant difference from the Q1 group; specifically, the Q4 group had a lower incidence of AMD. Likewise, the Q3 and Q4 groups for vitamin B2 displayed a negative correlation with AMD across models 1, 2, and 3, with statistical significance compared to the Q1 group. Additionally, the Q2 group for folic acid revealed a negative correlation with AMD in models 1, 2, and 3, also showing statistical significance from the Q1 group. These findings suggest that different levels of vitamin intake do not yield identical preventive effects against AMD (Table 2).

**Table 2:** Logistic regression model analysis.

Variables	Category	Model 1		Model 2		Model 3	
		OR (95%CI)	*P*	OR (95%CI)	*P*	OR (95%CI)	*P*
B1	Q1	Ref					
	Q2	0.55 (0.27–1.13)	0.11	0.45 (0.20–0.98)	0.06	0.45 (0.20–0.99)	0.07
	Q3	0.59 (0.27–1.29)	0.20	0.47 (0.21–1.05)	0.08	0.48 (0.21–1.09)	0.11
	Q4	0.47 (0.24–0.92)	0.03	0.56 (0.30–1.04)	0.08	0.58 (0.31–1.11)	0.13
B2	Q1	Ref					
	Q2	1.15 (0.54–2.42)	0.70	1.19 (0.53–2.67)	0.66	1.24 (0.56–2.71)	0.59
	Q3	0.40 (0.19–0.84)	0.02	0.38 (0.18–0.82)	0.02	0.39 (0.18–0.86)	0.04
	Q4	0.27 (0.12–0.64)	0.005	0.32 (0.13–0.77)	0.02	0.32 (0.13–0.80)	0.03
B6	Q1	Ref					
	Q2	1.27 (0.65–2.46)	0.48	1.11 (0.55–2.24)	0.75	1.12 (0.55–2.29)	0.75
	Q3	0.92 (0.38–2.19)	0.85	0.74 (0.30–1.81)	0.52	0.73 (0.29–1.79)	0.50
	Q4	0.54 (0.26–1.13)	0.11	0.60 (0.29–1.26)	0.19	0.62 (0.30–1.30)	0.24
B12	Q1	Ref					
	Q2	1.72 (0.77–3.84)	0.19	1.86 (0.76–4.57)	0.19	1.97 (0.76–5.11)	0.19
	Q3	0.89 (0.40–1.97)	0.78	0.85 (0.39–1.83)	0.68	0.82 (0.37–1.81)	0.64
	Q4	0.81 (0.40–1.62)	0.56	0.86 (0.38–1.94)	0.72	0.87 (0.39–1.97)	0.76
E	Q1	Ref					
	Q2	1.13 (0.48–2.63)	0.76	1.11 (0.45–2.68)	0.81	1.12 (0.46–2.70)	0.80
	Q3	1.49 (0.82–2.70)	0.19	1.54 (0.74–3.19)	0.25	1.53 (0.70–3.32)	0.30
	Q4	0.51 (0.23–1.15)	0.11	0.64 (0.28–1.44)	0.29	0.65 (0.29–1.46)	0.32
Folic acid	Q1	Ref					
	Q2	0.39 (0.17–0.87)	0.03	0.36 (0.15–0.86)	0.03	0.35 (0.14–0.84)	0.04
	Q3	0.53 (0.25–1.11)	0.10	0.54 (0.26–1.13)	0.12	0.51 (0.24–1.09)	0.11
	Q4	0.51 (0.22–1.19)	0.13	0.49 (0.21–1.10)	0.10	0.49 (0.21–1.14)	0.13

CI: Confidence interval.

### 3.3 Subgroup analysis

**Figure 2:**
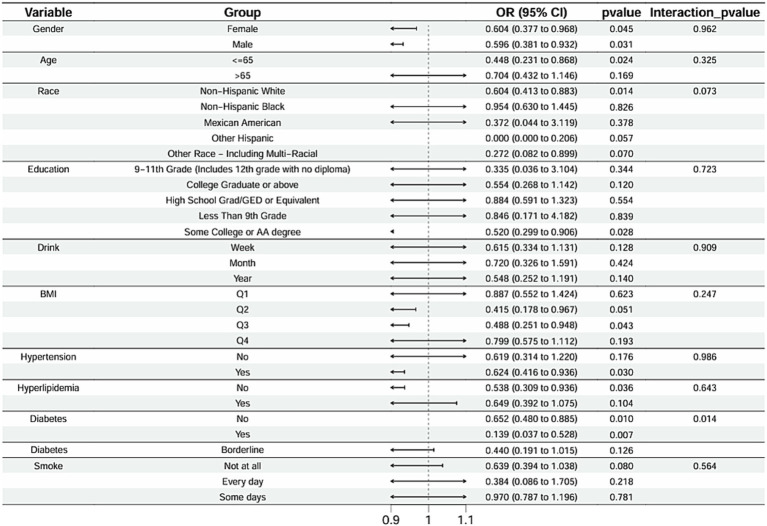
Vitamin B1 subgroup analysis.

**Figure 3:**
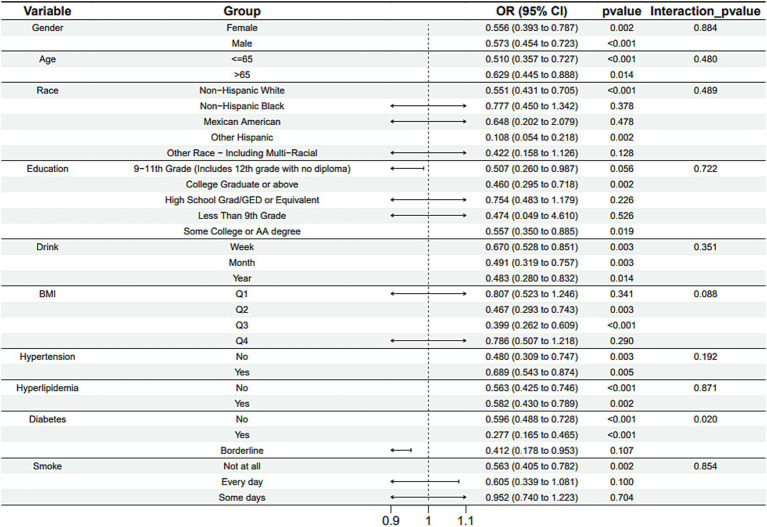
Vitamin B2 subgroup analysis.

**Figure 4:**
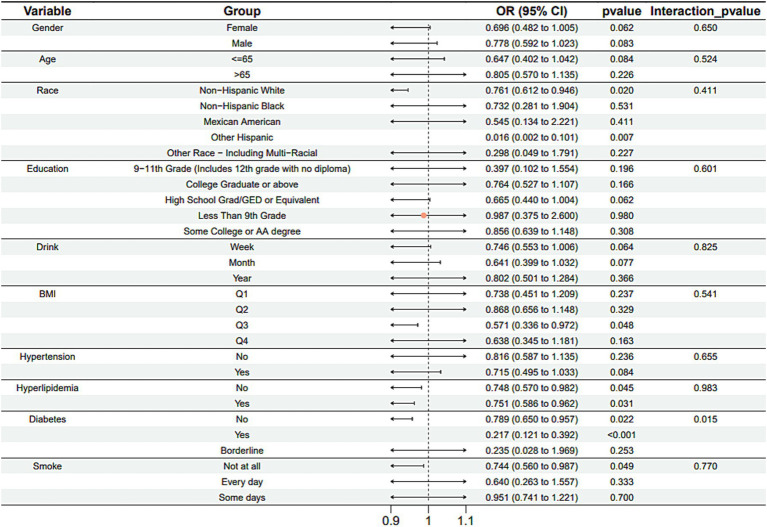
Vitamin B6 subgroup analysis.

**Figure 5:**
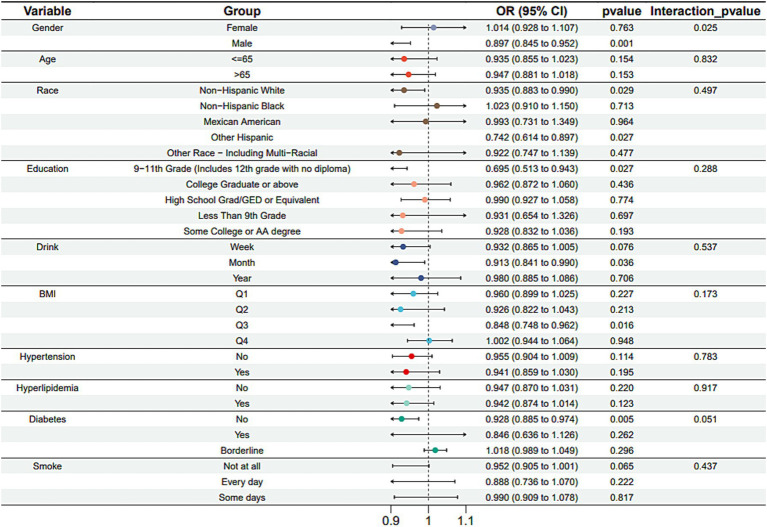
Vitamin B12 subgroup analysis.

**Figure 6:**
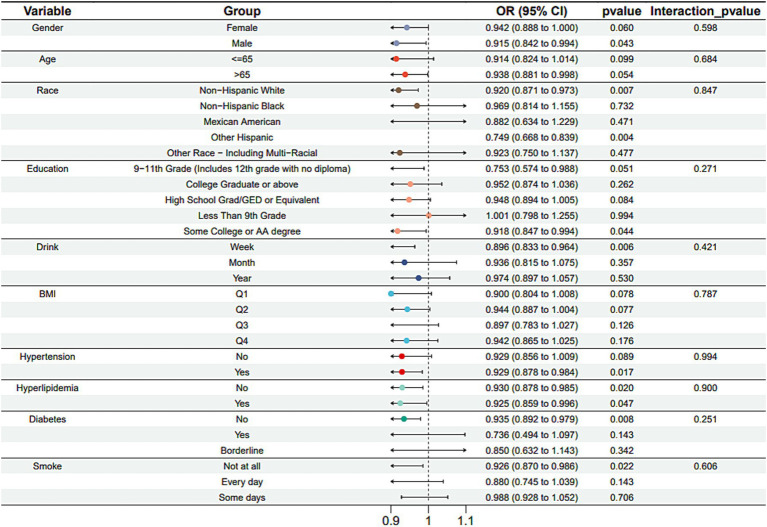
Vitamin E subgroup analysis.

**Figure 7:**
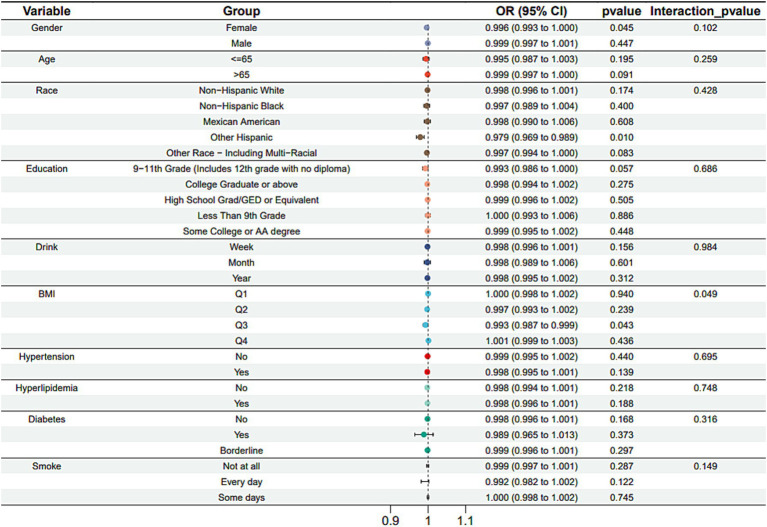
Folic acid subgroup analysis.

Subgroup analysis results showed that the causal association between vitamin B1, B2 and AMD in the gender group existed in both male and female groups. Additionally, the causal association between folic acid and AMD was present in the female group, whereas a causal association between vitamins B12 and E and AMD was noted exclusively in the male group. With the exception of vitamin B12, no significant interactions between vitamins and gender were identified. In age subgroups, a causal association between vitamin B1 and AMD was found in individuals aged 65 years or younger, whereas an association between vitamin B2 and AMD was observed in both age groups. No significant differences were noted for vitamins B6, folic acid, vitamin B12, and vitamin E between age groups, and there were no interactions between each vitamin and age. In the Non-Hispanic White population, causal associations were established between vitamins B1 and AMD. Causal associations involving vitamins B2, B12, B6 and E and AMD were present in both the Non-Hispanic White and Other Hispanic groups, whereas a causal association between folic acid and AMD was identified only in the Other Hispanic group, although no significant differences were observed between groups. Within the education subgroup, vitamin B1 and E were causally associated with AMD among individuals with Some College or an Associate degree. Vitamin B12 were causally associated with AMD among individuals with 9-11th Grade (Includes 12th grade with no diploma). Causal associations for vitamins B2 and AMD were found in both the Some College or Associate degree group and the College graduate or higher group, with no significant differences between the groups. Among the drinking habit groups, a causal association between vitamin B2 and AMD was found across all drinking habit categories. Additionally, a causal association between vitamin B12 and AMD was noted in the monthly drinking group, whereas an association between vitamin E and AMD was observed in the weekly drinking group, though no significant differences were present between groups. In the smoking habit subgroup, causal associations involving vitamins B2, B6, E, and AMD were observed in the Non-smoking group, yet no significant differences were noted between the groups. Among BMI quartile groups, a causal association between vitamin B2 and AMD was identified in the Q2 group, whereas vitamins B1, B2, B6, folic acid, and B12 were causally associated with AMD in the Q3 group, without significant differences between groups. In groups categorized by hypertension status, causal associations between vitamins B1 and E and AMD were found in the hypertensive group, whereas the causal association between vitamin B2 and AMD existed in both hypertensive and Non-hypertensive groups, but without significant differences. Conversely, in groups with and without diabetes, causal associations involving vitamins B1 and B2 and AMD were present in both diabetic and Non-diabetic groups, with statistical differences observed between these groups. Furthermore, a causal association between vitamin B6 and AMD was identified in the diabetes prevalence group, showing an interaction with diabetes. Lastly, causal associations between vitamins B12, E, and AMD were noted in the Non-diabetic group, with no significant differences between groups (Tables 3–8; Figures 2–7).

**Table 3:** Vitamin B1 subgroup analysis.

Variable	Group	β	Std. Error	*t* value	*P*	Interaction *P* value
Gender	Female	−0.50	0.24	−2.09	0.04	0.96
Gender	Male	−0.51	0.22	−2.26	0.03	
Age	<=65	−0.80	0.33	−2.37	0.02	0.32
Age	>65	−0.35	0.24	−1.41	0.16	
Race	Non-Hispanic White	−0.50	0.19	−2.60	0.01	0.07
Race	Non-Hispanic Black	−0.04	0.21	−0.22	0.82	
Race	Mexican American	−0.98	1.08	−0.91	0.37	
Race	Other Hispanic	−7.78	3.16	−2.45	0.05	
Race	Other race—including multi-racial	−1.30	0.61	−2.13	0.07	
Education	9-11th grade (includes 12th grade with no diploma)	−1.09	1.13	−0.96	0.34	0.72
Education	College graduate or above	−0.59	0.36	−1.60	0.12	
Education	High School Grad/GED or Equivalent	−0.12	0.20	−0.59	0.55	
Education	Less than 9th grade	−0.16	0.81	−0.20	0.83	
Education	Some college or AA degree	−0.65	0.28	−2.30	0.02	
Drink	Week	−0.48	0.31	−1.56	0.12	0.90
Drink	Month	−0.32	0.40	−0.81	0.42	
Drink	Year	−0.60	0.39	−1.51	0.13	
BMI	Q1	−0.12	0.24	−0.49	0.62	0.24
BMI	Q2	−0.87	0.43	−2.03	0.05	
BMI	Q3	−0.71	0.33	−2.11	0.04	
BMI	Q4	−0.22	0.16	−1.33	0.19	
Hypertension	No	−0.47	0.34	−1.38	0.17	0.98
Hypertension	Yes	−0.47	0.20	−2.28	0.02	
Hyperlipidemia	No	−0.62	0.28	−2.19	0.03	0.64
Hyperlipidemia	Yes	−0.43	0.25	−1.6	0.10	
Diabetes	No	−0.42	0.15	−2.74	0.01	0.01
Diabetes	Yes	−1.97	0.68	−2.89	0.006	
Diabetes	Borderline	−0.81	0.42	−1.92	0.12	
Smoke	Not at all	−0.44	0.24	−1.81	0.08	0.56
Smoke	Every day	−0.95	0.76	−1.25	0.21	
Smoke	Some days	−0.03	0.10	−0.28	0.78	

SD: standard error.

**Table 4:** Vitamin B2 subgroup analysis.

Variable	Group	β	Std. Error	*t* value	*P*	Interaction *P* value
Gender	Female	−0.58	0.17	−3.31	0.002	0.88
Gender	Male	−0.55	0.11	−4.69	5.58E-05	
Age	<=65	−0.67	0.18	−3.71	0.0008	0.48
Age	>65	−0.46	0.17	−2.63	0.01	
Race	Non-Hispanic White	−0.59	0.12	−4.75	5.07E-05	0.48
Race	Non-Hispanic Black	−0.25	0.27	−0.90	0.37	
Race	Mexican American	−0.43	0.59	−0.72	0.47	
Race	Other Hispanic	−2.22	0.35	−6.24	0.001	
Race	Other race—including multi-racial	−0.86	0.50	−1.72	0.12	
Education	9-11th grade (includes 12th grade with no diploma)	−0.67	0.33	−1.99	0.05	0.72
Education	College Graduate or above	−0.77	0.22	−3.41	0.001	
Education	High school grad/GED or equivalent	−0.28	0.22	−1.23	0.22	
Education	Less than 9th grade	−0.74	1.16	−0.64	0.52	
Education	Some college or AA degree	−0.58	0.23	−2.47	0.01	
Drink	Week	−0.39	0.12	−3.28	0.002	0.35
Drink	Month	−0.71	0.22	−3.22	0.003	
Drink	Year	−0.72	0.27	−2.61	0.01	
BMI	Q1	−0.21	0.22	−0.96	0.34	0.08
BMI	Q2	−0.76	0.23	−3.20	0.003	
BMI	Q3	−0.91	0.21	−4.26	0.0002	
BMI	Q4	−0.24	0.22	−1.07	0.29	
Hypertension	No	−0.73	0.22	−3.25	0.002	0.19
Hypertension	Yes	−0.37	0.12	−3.06	0.004	
Hyperlipidemia	No	−0.57	0.14	−4.00	0.0003	0.87
Hyperlipidemia	Yes	−0.54	0.15	−3.49	0.001	
Diabetes	No	−0.51	0.10	−5.05	1.99E-05	0.01
Diabetes	Yes	−1.28	0.26	−4.85	3.48E-05	
Diabetes	Borderline	−0.88	0.42	−2.07	0.10	
Smoke	Not at all	−0.57	0.16	−3.42	0.001	0.85
Smoke	Every day	−0.50	0.29	−1.69	0.10	
Smoke	Some days	−0.04	0.12	−0.38	0.70	

SD: standard error.

**Table 5:** Vitamin B6 subgroup analysis.

Variable	Group	β	Std. Error	*t* value	*P*	Interaction *P* value
Gender	Female	−0.36	0.18	−1.93	0.06	0.64
Gender	Male	−0.25	0.13	−1.79	0.08	
Age	<=65	−0.43	0.24	−1.78	0.08	0.52
Age	>65	−0.21	0.17	−1.23	0.22	
Race	Non-Hispanic White	−0.27	0.11	−2.46	0.01	0.41
Race	Non-Hispanic Black	−0.31	0.48	−0.64	0.53	
Race	Mexican American	−0.60	0.71	−0.84	0.41	
Race	Other Hispanic	−4.14	0.94	−4.38	0.007	
Race	Other race—including multi-racial	−1.21	0.91	−1.32	0.22	
Education	9-11th grade (includes 12th grade with no diploma)	−0.92	0.69	−1.32	0.19	0.60
Education	College graduate or above	−0.26	0.18	−1.42	0.16	
Education	High school grad/GED or equivalent	−0.40	0.21	−1.94	0.06	
Education	Less than 9th grade	−0.01	0.49	−0.02	0.97	
Education	Some college or AA degree	−0.15	0.14	−1.03	0.30	
Drink	Week	−0.29	0.15	−1.92	0.06	0.82
Drink	Month	−0.44	0.24	−1.83	0.07	
Drink	Year	−0.22	0.24	−0.91	0.36	
BMI	Q1	−0.30	0.25	−1.20	0.23	0.54
BMI	Q2	−0.14	0.14	−0.99	0.32	
BMI	Q3	−0.55	0.27	−2.06	0.04	
BMI	Q4	−0.44	0.31	−1.42	0.16	
Hypertension	No	−0.20	0.16	−1.20	0.23	0.65
Hypertension	Yes	−0.33	0.18	−1.78	0.08	
Hyperlipidemia	No	−0.29	0.13	−2.09	0.04	0.98
Hyperlipidemia	Yes	−0.28	0.12	−2.26	0.03	
Diabetes	No	−0.23	0.09	−2.40	0.02	0.01
Diabetes	Yes	−1.52	0.30	−5.07	1.86E-05	
Diabetes	Borderline	−1.44	1.08	−1.33	0.25	
Smoke	Not at all	−0.29	0.14	−2.04	0.04	0.77
Smoke	Every day	−0.44	0.45	−0.98	0.33	
Smoke	Some days	−0.05	0.12	−0.39	0.69	

SD: standard error.

**Table 6:** Vitamin B12 subgroup analysis.

Variable	Group	β	Std. Error	*t* value	*P*	Interaction *P* value
Gender	Female	0.01	0.04	0.30	0.76	0.02
Gender	Male	−0.10	0.03	−3.57	0.001	
Age	<=65	−0.06	0.04	−1.46	0.15	0.83
Age	>65	−0.05	0.03	−1.46	0.15	
Race	Non-Hispanic White	−0.06	0.02	−2.29	0.02	0.49
Race	Non-Hispanic Black	0.02	0.05	0.37	0.71	
Race	Mexican American	−0.007	0.15	−0.04	0.96	
Race	Other Hispanic	−0.29	0.09	−3.08	0.02	
Race	Other race—including multi-racial	−0.08	0.10	−0.75	0.47	
Education	9-11th grade (includes 12th grade with no diploma)	−0.36	0.15	−2.33	0.02	0.28
Education	College graduate or above	−0.03	0.04	−0.78	0.43	
Education	High school grad/GED or equivalent	−0.009	0.03	−0.28	0.77	
Education	Less than 9th grade	−0.07	0.18	−0.39	0.69	
Education	Some college or AA degree	−0.07	0.05	−1.33	0.19	
Drink	Week	−0.07	0.03	−1.84	0.07	0.53
Drink	Month	−0.09	0.04	−2.19	0.03	
Drink	Year	−0.01	0.05	−0.38	0.70	
BMI	Q1	−0.04	0.03	−1.23	0.22	0.17
BMI	Q2	−0.07	0.06	−1.27	0.21	
BMI	Q3	−0.16	0.06	−2.57	0.01	
BMI	Q4	0.002	0.03	0.06	0.94	
Hypertension	No	−0.04	0.02	−1.62	0.11	0.78
Hypertension	Yes	−0.06	0.04	−1.32	0.19	
Hyperlipidemia	No	−0.05	0.04	−1.25	0.21	0.91
Hyperlipidemia	Yes	−0.06	0.03	−1.58	0.12	
Diabetes	No	−0.07	0.02	−3.03	0.004	0.05
Diabetes	Yes	−0.16	0.14	−1.14	0.26	
Diabetes	Borderline	0.01	0.01	1.20	0.29	
Smoke	Not at all	−0.04	0.02	−1.91	0.06	0.43
Smoke	Every day	−0.11	0.09	−1.24	0.22	
Smoke	Some days	−0.01	0.04	−0.23	0.81	

SD: standard error.

**Table 7:** Vitamin E subgroup analysis.

Variable	Group	β	Std. Error	*t* value	*P*	Interaction *P* value
Gender	Female	−0.05	0.03	−1.95	0.05	0.59
Gender	Male	−0.08	0.04	−2.10	0.04	
Age	<=65	−0.08	0.05	−1.70	0.09	0.68
Age	>65	−0.06	0.03	−2.00	0.05	
Race	Non-Hispanic White	−0.08	0.02	−2.93	0.006	0.84
Race	Non-Hispanic Black	−0.03	0.08	−0.34	0.73	
Race	Mexican American	−0.12	0.16	−0.74	0.47	
Race	Other Hispanic	−0.28	0.05	−4.97	0.004	
Race	Other race—including multi-racial	−0.07	0.10	−0.75	0.47	
Education	9-11th grade (includes 12th grade with no diploma)	−0.28	0.13	−2.04	0.05	0.27
Education	College graduate or above	−0.04	0.04	−1.14	0.26	
Education	High school grad/GED or equivalent	−0.05	0.02	−1.79	0.08	
Education	Less than 9th grade	0.0009	0.11	0.008	0.99	
Education	Some college or AA degree	−0.08	0.04	−2.10	0.04	
Drink	Week	−0.11	0.03	−2.95	0.005	0.42
Drink	Month	−0.06	0.07	−0.93	0.35	
Drink	Year	−0.02	0.04	−0.63	0.52	
BMI	Q1	−0.10	0.05	−1.82	0.07	0.78
BMI	Q2	−0.05	0.03	−1.83	0.07	
BMI	Q3	−0.10	0.06	−1.57	0.12	
BMI	Q4	−0.06	0.04	−1.38	0.17	
Hypertension	No	−0.07	0.04	−1.75	0.08	0.99
Hypertension	Yes	−0.07	0.02	−2.52	0.01	
Hyperlipidemia	No	−0.07	0.02	−2.45	0.01	0.89
Hyperlipidemia	Yes	−0.07	0.03	−2.06	0.04	
Diabetes	No	−0.06	0.02	−2.85	0.007	0.25
Diabetes	Yes	−0.30	0.20	−1.50	0.14	
Diabetes	Borderline	−0.16	0.15	−1.07	0.34	
Smoke	Not at all	−0.07	0.03	−2.41	0.02	0.60
Smoke	Every day	−0.12	0.08	−1.50	0.14	
Smoke	Some days	−0.01	0.03	−0.38	0.70	

SD: standard error.

**Table 8:** Folic acid subgroup analysis.

Variable	Group	β	Std. Error	*t* value	*P*	Interaction *P* value
Gender	Female	−0.003	0.001	−2.09	0.04	0.10
Gender	Male	−0.0008	0.001	−0.77	0.44	
Age	<=65	−0.005	0.004	−1.32	0.19	0.25
Age	>65	−0.001	0.0007	−1.74	0.09	
Race	Non-Hispanic White	−0.001	0.001	−1.39	0.17	0.42
Race	Non-Hispanic Black	−0.003	0.003	−0.86	0.40	
Race	Mexican American	−0.002	0.004	−0.52	0.60	
Race	Other Hispanic	−0.02	0.005	−3.99	0.01	
Race	Other race—including multi-racial	−0.003	0.001	−2.01	0.08	
Education	9-11th grade (includes 12th grade with no diploma)	−0.007	0.003	−1.99	0.05	0.68
Education	College graduate or above	−0.002	0.001	−1.11	0.27	
Education	High school grad/GED or equivalent	−0.001	0.001	−0.67	0.50	
Education	Less than 9th grade	−0.0004	0.003	−0.14	0.88	
Education	Some college or AA degree	−0.001	0.001	−0.76	0.44	
Drink	Week	−0.001	0.001	−1.45	0.15	0.98
Drink	Month	−0.002	0.004	−0.52	0.60	
Drink	Year	−0.001	0.001	−1.02	0.31	
BMI	Q1	−8.96E-05	0.001	−0.07	0.93	0.04
BMI	Q2	−0.002	0.002	−1.20	0.23	
BMI	Q3	−0.006	0.003	−2.12	0.04	
BMI	Q4	0.0008	0.001	0.78	0.43	
Hypertension	No	−0.001	0.001	−0.78	0.43	0.69
Hypertension	Yes	−0.002	0.001	−1.51	0.13	
Hyperlipidemia	No	−0.002	0.001	−1.25	0.21	0.74
Hyperlipidemia	Yes	−0.001	0.001	−1.34	0.188	
Diabetes	No	−0.001	0.001	−1.41	0.16	0.31
Diabetes	Yes	−0.01	0.01	−0.90	0.37	
Diabetes	Borderline	−0.001	0.001	−1.19	0.29	
Smoke	Not at all	−0.001	0.001	−1.08	0.28	0.14
Smoke	Every day	−0.008	0.005	−1.59	0.12	
Smoke	Some days	−0.0003	0.001	−0.33	0.74	

SD: standard error.

### 3.4 Multicollinearity regression analysis

This study performed a multicollinearity regression analysis involving B vitamins, vitamin E, and various confounding factors to further validate the stability of the results. The findings indicated that there was no significant multicollinearity among B vitamins, vitamin E, and the confounding factors (Table 9).

**Table 9:** Multicollinearity regression analysis.

Variable	Vif_values	Df	Tolerance_values
Vitamin B1	1.08	1.00	0.92
Age	1.33	1.00	0.74
Gender	1.12	1.00	0.89
Race	1.38	4.00	0.72
Education	1.37	4.00	0.72
BMI	1.22	1.00	0.81
Smoke	1.30	2.00	0.76
Drink	1.14	2.00	0.87
Hypertension	1.15	1.00	0.86
Hyperlipidemia	1.04	1.00	0.95
Diabetes	1.13	2.00	0.88
Vitamin B2	1.12	1.00	0.89
Age	1.33	1.00	0.74
Gender	1.12	1.00	0.88
Race	1.42	4.00	0.70
Education	1.36	4.00	0.73
BMI	1.22	1.00	0.81
Smoke	1.30	2.00	0.76
Drink	1.14	2.00	0.87
Hypertension	1.16	1.00	0.86
Hyperlipidemia	1.05	1.00	0.95
Diabetes	1.13	2.00	0.88
Vitamin B6	1.10	1.00	0.90
Age	1.34	1.00	0.74
Gender	1.12	1.00	0.88
Race	1.37	4.00	0.72
Education	1.37	4.00	0.72
BMI	1.22	1.00	0.81
Smoke	1.30	2.00	0.76
Drink	1.14	2.00	0.87
Hypertension	1.15	1.00	0.86
Hyperlipidemia	1.04	1.00	0.95
Diabetes	1.13	2.00	0.87
Vitamin B12	1.04	1.00	0.95
Age	1.32	1.00	0.75
Gender	1.09	1.00	0.91
Race	1.37	4.00	0.72
Education	1.36	4.00	0.73
BMI	1.22	1.00	0.81
Smoke	1.30	2.00	0.76
Drink	1.14	2.00	0.87
Hypertension	1.15	1.00	0.86
Hyperlipidemia	1.05	1.00	0.95
Diabetes	1.14	2.00	0.87
Folic acid	1.03	1.00	0.96
Age	1.32	1.00	0.75
Gender	1.07	1.00	0.92
Race	1.38	4.00	0.72
Education	1.37	4.00	0.72
BMI	1.22	1.00	0.81
Smoke	1.30	2.00	0.76
Drink	1.14	2.00	0.87
Hypertension	1.15	1.00	0.86
Hyperlipidemia	1.04	1.00	0.95
Diabetes	1.13	2.00	0.88
Vitamin E	1.06	1.00	0.93
Age	1.33	1.00	0.74
Gender	1.07	1.00	0.92
Race	1.38	4.00	0.72
Education	1.37	4.00	0.72
BMI	1.22	1.00	0.81
Smoke	1.31	2.00	0.76
Drink	1.14	2.00	0.87
Hypertension	1.15	1.00	0.86
Hyperlipidemia	1.05	1.00	0.94
Diabetes	1.13	2.00	0.88

VIF: variance inflation factor.

## 4 Discussion

The objective of this study was to determine an independent association between vitamin intake and AMD. By utilizing two cycles of the NHANES data from 2005–2006 and 2007–2008 and controlling for several potential confounding variables, we found that individuals in the United States with high vitamin intakes B (1, 2, 6, and 12), E and folic acid were less likely to be diagnosed with AMD. The preventive effects of vitamin B2 and folic acid were especially notable.

According to clinical presentation, the Age-Related Eye Disease Study team classifies AMD into four stages: AMD-free, early, middle, and advanced. In the advanced stages, two distinct manifestations emerge (24). The first is the development of confluent areas of atrophy affecting photoreceptors and the retinal pigment epithelium, known as geographic atrophy (atrophic AMD). The second is the proliferation of abnormal blood vessels in the macular region, referred to as neovascular AMD (exudative AMD) (24). Neovascular AMD necessitates repeated intravitreal injections of anti-vascular endothelial growth factor (VEGF) drugs, whereas there is currently no clinical treatment available that can slow progression or restore vision in cases of geographic atrophy (24). Thus, effective prevention and early detection methods are essential for mitigating AMD-related blindness in the elderly population.

Oxidative stress is a significant factor contributing to the development of AMD (25). The retina is a tissue with a high oxygen demand, even exceeding that of the brain by weight. The local oxygen metabolic environment in the retina is critical for maintaining a balance between oxygen supply and demand. Retinal phototransduction is an ongoing process that converts light into vision, resulting in low energy consumption and the production of reactive oxygen species (ROS) as metabolic byproducts. Under normal circumstances, ROS facilitate regular cell metabolism; however, oxidative stress occurs when the levels of ROS exceed the capacity of the antioxidant system (26). Retinal tissue, particularly the retinal pigment epithelium (RPE) cell layer in the macula, is likely to generate excessive exogenous ROS in a high-oxygen environment. Elevated ROS levels can lead to oxidative damage to proteins, lipids, and mitochondrial DNA (27). During mitochondrial damage, certain proteins are released into the cytoplasm due to the permeabilization of the mitochondrial outer membrane, triggering cell death (28). Increasing research efforts are directed at understanding the processes that lead to RPE cell death under oxidative stress conditions (29, 30). Advanced AMD, especially geographic atrophy, is characterized by the loss of RPE cells and subsequent dysfunction of photoreceptors (24). In summary, oxidative stress is a critical factor in the pathogenesis of AMD.

This study found that the intake of vitamins B and E was negatively correlated with the prevalence of AMD. As an antioxidant, vitamin E slows the progression of AMD by inhibiting oxidative stress (31), which may explain the observed results. The potential of B vitamins to delay the onset of AMD may be attributed to several factors: B vitamins are crucial for DNA methylation, synthesis, repair, and replication (32) and they are involved in one-carbon metabolism. A deficiency in B vitamins could impair DNA methylation, leading to one-carbon metabolism disorders that may increase the risk of age-related neurodegenerative diseases (32, 33). Additionally, B vitamins help regulate plasma homocysteine levels in the body. Without B vitamin cofactors, homocysteine can build up, affecting amino acid and nucleotide metabolism (9). Elevated homocysteine is linked to an increased risk of neurodegenerative and cardiovascular diseases and is also associated with AMD (34). This association is complex and may be influenced by different states of the three B vitamin cofactors, other B vitamins, or common variants of methylenetetrahydrofolate reductase, a key enzyme in converting homocysteine to methionine (35). Furthermore, vitamin B1 acts as an antioxidant, similar to vitamin E, and may slow AMD progression by mitigating oxidative stress (36).

An overlap of confidence intervals was observed in the results. Based on previous research (35), the reason for this phenomenon may be that the amount of vitamin intake required at each age. This study categorized B vitamins and vitamin E into quartiles and conducted a logistic regression analysis. The findings indicated that the Q4 group of vitamin B1 was negatively correlated with AMD in model 1 and statistically differed from the Q1 group, suggesting that the Q4 group experienced a lower incidence of AMD. Similarly, the Q3 and Q4 groups of vitamin B2 exhibited a negative correlation with AMD across models 1, 2, and 3, and were statistically different from the Q1 group, again indicating a lower incidence of AMD in the Q4 group. The Q2 group of folic acid also showed a negative correlation with AMD in these models and was statistically different from the Q1 group, indicating a lower incidence of AMD in the Q2 group. These results suggest that after categorizing B vitamins and vitamin E into quartiles and conducted a logistic regression analysis, the effects of vitamins remain significant. The findings align with previous studies affirming the importance of B vitamin intake in the development of AMD (36).

Some differences were also observed in the subgroup analysis, which may be related to intestinal microbial changes and dietary habits (37). The adult intestines host a complex microbial ecosystem comprising an estimated 100 trillion organisms (Host–Bacterial Mutualism in the Human Intestine) (38). Numerous beneficial effects of gut commensals have been reported, including facilitating nutrient metabolism, maintaining the intestinal barrier, and preventing pathologic bacterial colonies from establishing themselves (38). The gut microbiota plays a crucial role in digestion, vitamin production, and the synthesis of short-chain fatty acids (SCFAs) such as acetate, propionate, and butyrate (39). A research study indicated that individuals with advanced AMD exhibited signs of intestinal dysbiosis, characterized by a distinct composition of gut bacteria compared to healthy older adults. Specifically, the bacterial genera Anaerotruncus and Oscillibacter, along with the species *Ruminococcus torques* and *Eubacterium ventriosum*, were found to be differentially abundant (39). Conversely, AMD subjects exhibited reduced levels of Oscillospira, Blautia, and Dorea compared to their healthy counterparts (40). In the pathogenesis of AMD, regulated immune activation involving the recruitment of microglia and macrophages to the subretinal and choroidal regions, mast cell activation, and immune responses from the retinal pigment epithelium (RPE) are considered key factors (41). The gut microbiota may potentially modulate inflammation and microglial function during the development and progression of AMD (39). Vitamin B1 is absorbed in the duodenum and converted, with magnesium as a cofactor, to its active form, thiamine pyrophosphate (TPP) (42). TPP acts as a cofactor at crucial steps in the citric acid cycle and the pentose phosphate pathway (42). TPP also plays a major role in the aerobic metabolism of glucose for energy production (42). Certain intestinal microbiota, including *Bacteroides fragilis*, Prevotella, *Fusobacterium* var*ium*, Actinobacteria, and Clostridium, can produce vitamin B1 (43). About half of the enzymes present in these microbiotas are involved in the *de novo* synthesis of vitamin B1 (44). However, some gut microbiota lack the vitamin B1 synthesis pathway and require an external source for growth, such as Ruminococcaceae (44). This finding indicates that vitamin B1 intake can influence the composition of the gut microbiota. The active forms of vitamin B2 are essential for synthesizing niacin, folic acid, vitamin B6, and all heme proteins. Additionally, vitamin B2 is necessary for the metabolism of carbohydrates, proteins, and fats into glucose. Its antioxidant effects are crucial for cellular respiration and play a key role in immune system function (45). Vitamin B2 also influences the composition of the gut microbiome. Replenishment of vitamin B2 has been reported to alter the composition and diversity of gut microbiota in mice (46). In clinical interventions, vitamin B2 supplementation for two weeks increased the relative abundance of *F. prausnitzii* in healthy individuals (47). Moreover, treatment with vitamins B2 and C significantly notably the number of Proteobacteria while showing a trend toward an increase in Firmicutes and a decrease in Bacteroidetes (48). The active form of vitamin B6 is a coenzyme that supports numerous enzymes in performing various functions, including the maintenance of normal homocysteine levels, supporting immune function and brain health, and the breakdown of carbohydrates, proteins, and fats (49). Vitamin B6 also affects the growth of gut microbiota (50). Unfortunately, investigations about this topic are limited. Folic acid is crucial for nucleic acid synthesis and red blood cell production. It plays a role in converting homocysteine to methionine, which is essential for hematopoiesis and the prevention of megaloblastic anemia (51). Folic acid deficiency can impact bacterial diversity. In a study involving gnotobiotic mice, a folic acid-deficient diet increased microbial diversity after a 21-day treatment compared to a micronutrient-sufficient diet. However, a 14-day full diet treatment did not alter this trend (52). Another study assessed fecal microbiota composition based on human gut microbiota. The fecal microbiota community shows lower diversity in healthy volunteers on a diet low in folic acid. Additionally, the fecal microbiota has a higher potential to produce folic acid in *in vitro* experiments (53). This finding suggests that folic acid deficiency may decrease the richness of human gut microbiota. Vitamin B12 is required for red blood cell production, neurologic function, and myelin synthesis. This vitamin serves as a cofactor in DNA and RNA synthesis, as well as in the synthesis and metabolism of hormones, proteins, and lipids (49). In humans, vitamin B12 may increase the proportion of Proteobacteria and Verrucomicrobia (54) while reducing the abundance of Bacteroidetes (50). Vitamin E possesses strong antioxidant and anti-inflammatory properties (55). Research findings indicate that colon-targeted Vitamin E (−tocopherol, 100 mg/kg) supplementation increased the production of SCFAs and the relative abundance of beneficial microorganisms such as Akkermansia, Lactobacillus, Bifidobacterium, and Faeca libacterium (48). In addition to affecting the composition of the gut microbiota, the intake of B vitamins and vitamin E may influence SCFA metabolism by regulating gut microbiota. Subsequently, SCFAs impact neuroinflammation by altering glial cell morphology, continuously modulating microglia maturation, and regulating levels of neurotrophic factors, which enhances neurogenesis and improves neuronal homeostasis and function, further affecting AMD (56). Based on the above studies, we hypothesize that B vitamins and vitamin E may also affect AMD by regulating gut microbiome composition. However, differences in results may arise in subgroup analysis because the effects of each vitamin on the gut microbiome are not identical. Additionally, differences in race, age, and gender appear to influence the results of these analyses. Since the content of each vitamin varies across different dietary habits (37), and the amounts of vitamins required by individuals of different races and genders at various age stages are not the same, these factors may also contribute to the differences observed in subgroup analysis.

Some studies have reported a negative correlation between the intake of vitamins A, C, and D and the incidence of AMD (9, 30, 57, 58), which contrasts with the findings of the present study. The “Age-Related Eye Disease Study (AREDS)” was a significant multicenter prospective investigation of eye diseases, including AMD, conducted by the NIH in the United States (5, 15). The findings suggest that oral antioxidant supplements containing vitamin C, vitamin E, and provitamin A carotenoids may slow the progression of AMD. In a related multicenter, double-blind, randomized placebo-controlled trial, Li et al. (59) reported results that align with these findings. Additionally, Millen et al. (16) performed a prospective analysis involving a subset of participants (*n* = 1,225) from the Atherosclerosis Risk in Communities Study, indicating that high concentrations of 25(OH)D may be associated with lower odds of developing early AMD. The study by Kabatas et al. (60) found significantly reduced levels of 25(OH) vitamin D in individuals with advanced-stage AMD.

The discrepancies among these studies may arise from variations in sample size, ethnicity, and the confounding factors accounted for in each study. In the AREDS, 4,757 participants aged 55 to 80 years were recruited from 11 retinal specialty clinics across the United States between 1992 and 1998. In the AREDS2, 4,203 participants aged 50 to 85 years were enrolled from 82 retinal specialty clinics between 2006 and 2008 (61). The participants in Li’s study (59) were Chinese individuals diagnosed with AMD based on the classification system used in the Age-Related Eye Disease Study. The Atherosclerosis Risk in Communities Study (16) is a population-based, prospective investigation on atherosclerosis that has taken place since 1987–1989, involving participants aged 45 to 65 years at the initial visit and recruited from four centers: Forsyth County, North Carolina; Jackson, Mississippi; Minneapolis, Minnesota; and Washington County, Maryland. Participants in Kabatas’s study (60) were Turkish and were excluded if they had received immunomodulator treatment, had chronic renal, hepatic, or parathyroid diseases, had received intravitreal anti-VEGF injections within the previous month, or were undergoing antioxidant or vitamin D treatment. The NHANES consists of a series of cross-sectional interviews and examinations of the civilian, noninstitutionalized population of the United States, providing data on AMD only from 2005 to 2008. Moreover, the exclusion criteria applied in this study differed from those in prior research, contributing to the variations observed in the results.

This study has several limitations. First, as a cross-sectional study, it cannot establish a causal connection between vitamin consumption levels and AMD; instead, it can only suggest associations. Second, since our study focused exclusively on older Americans, the findings may not be generalizable to other populations. Further randomized clinical trials are needed to confirm these findings. However, our study also possesses notable strengths. Data were collected from a diverse and representative sample, enhancing the generalizability of the findings to the broader population of older Americans. Additionally, this research provides a comprehensive investigation into the association between vitamin intake and AMD within the U.S. population, contributing to its overall credibility.

## 5 Conclusion

This study found that higher vitamin intakes B and E were associated with a lower prevalence of AMD in the U.S. population. Eating a healthy diet rich in vitamins B and E, particularly B2 (eggs, green vegetables, meat, mushrooms, and almonds) may help to reduce vision loss due to AMD. However, since this is a cross-sectional study, causal associations between vitamin intake and AMD cannot be established. Further randomized clinical trials are needed to confirm these findings.

## Data Availability

The original contributions presented in the study are included in the article/supplementary material, further inquiries can be directed to the corresponding author.
